# Complete mitochondrial genome sequence and annotation of *Rhinogobius lentiginis* (Gobiiformes: Gobiidae: Gobionellinae)

**DOI:** 10.1080/23802359.2023.2189497

**Published:** 2023-03-20

**Authors:** Lin Song, Xiao Jiang Chen, Yi Wen Gu, Quan Wang

**Affiliations:** Jiangsu Agri-Animal Husbandry Vocational College, Taizhou, Jiangsu Province, P. R.China

**Keywords:** Mitochondrial genome, *Rhinogobius lentiginis*, Gobionellinae, phylogenetic analysis

## Abstract

We report the complete mitochondrial genome of *Rhinogobius lentiginis*, which was found to be a circular molecule of 16,633 bp in length and included 13 protein-coding genes (PCGs), 2 rRNA genes, 22 tRNA genes, and a non-coding control region. The overall base composition was 28.44% A, 26.21% T, 16.33% G, and 29.02% C. Phylogenetic analyses using maximum-likelihood and Bayesian inference methods revealed a close genome relationship among *R. lentiginis*, *R. niger*, *R. shennongensis* and *R. maculagenys*. The complete mitogenome of *R. lentiginis* will provide a valuable resource for species classification and conservation.

## Introduction

*Rhinogobius lentiginis* (Gobiiformes: Gobiidae: Gobionellinae), an endemic species in China, is mainly distributed in the Lingjiang Basin, the Tingpangxi Basin, lower reaches of the Yangtze Basin and the Cao’ejiang River, which is a tributary of the Qiantang River Basin. *R. lentiginis* is a benthic, land-locked species that prefers slow-flowing water environment with fine gravel as the sediment. Human activities, such as the discharge of domestic and industrial sewage, stream dredging operations, and the construction of dams and reservoirs, will have a great impact on the survival of the goby. Taking the Qiantang River basin as an example, it was reported that there were 11 species of goby in 2011, but several of them were extremely rare. The population of these rare species is likely to decline or even become extinct due to the continuous deterioration of the basin environment (Li 2011). To develop strategies for the management of fisheries resources, the taxonomic survey and research on stream fish will be the primary work. Mitochondrial DNA is considered to be one of the most efficient and reliable molecular marker for species identification, evolutionary biology, population genetics and conservation biology (Ko et al. 2018). Therefore, we sequenced the complete mitogenome of *R. lentiginis* to present basic genetic information.

## Materials

**Figure 1.**
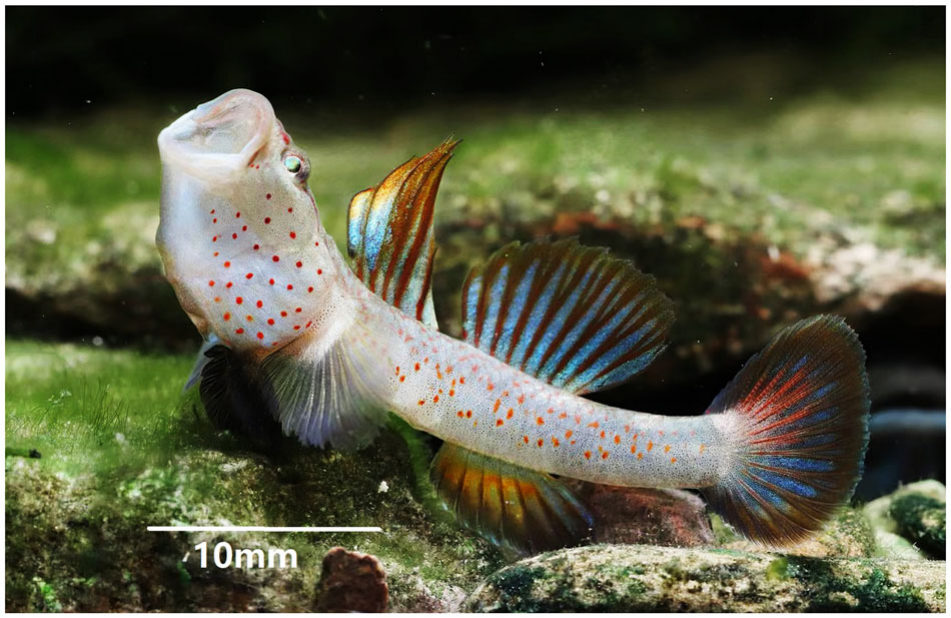
The specimen of *Rhinogobius lentiginis* from Lingjiang River, Linhai City, Zhejiang Province, China. The main identifiable morphological features are the first dorsal fin VI; the second dorsal fin I, 8; gluteal fin I, 7–8 (mainly 7); pectoral fin 14–15; longitudinal scales 30–32; transverse scales 10–11; dorsal fin anterior scale 0; vertebrae number 26–27 (mainly 27); complete type of sensory canal pores (Li 2011). (Photo by Lin Song).

A specimen of *R. lentiginis* (Figure 1) was captured by the fish traps from Lingjiang River, Linhai City, Zhejiang Province, China (28°50′52.06″N, 121°09′23.32″E), in April 2022, and was euthanized with tricaine methanesulfonate (MS-222, MilliporeSigma, St. Louis, MO, USA). The specimen was given voucher number ASTIH-21b1108d24 and preserved in 95% ethanol at the Aquatic Science and Technology Institution Herbarium (https://www.jsahvc.edu.cn/, Lin Song, tianxinlinger@126.com).

## Methods

Genomic DNA was isolated from muscle tissue using the Tguide Cell/tissue genomic DNA Extraction Kit (OSR-M401, Tiangen, Beijing, China), followed by quality control to ensure sample concentration, purity and integrity using the NanoDrop 2000 (Thermo Fisher Scientific, USA). A DNA library consisting of all sheared mitochondrial DNA was prepared using the MGIEasy DNA Library Prep Kit (MGI Technology, Shenzhen, China). The purificaiton and size selection of the library was completed using Agencourt SPRIselect (Beckman Coulter, USA). The Agilent 2100 Bioanalyzer (Agilent Technologies, USA) was used to check the quality of the quality of the library. Finally, the constructed library was sequenced on the Illumina HiSeq 4000 Sequencing platform (Illumina, CA, USA) with paired-end reads (150 bp).

The sequencing reads were assessed for quality using FastQC (http://www.bioinformatics.babraham.ac.uk/projects/fastqc), and low-quality reads as well as adapter sequences were trimmed using Trimmomatic ver. 0.40 (Bolger et al. 2014). The clean reads were mapped to the reference mitogenome of *Rhinogobius brunneus* (KT601096) using BWA v.0.7.17 (Li and Durbin 2009) with default parameters. Samtool v.1.9 (Li and Durbin 2009) was employed to retrieve the aligned mitochondrial reads. Then the retrieved reads were assembled with MetaSPAdes 3.13.0 (Nurk et al. 2017). The complete mitochondrial genome annotation was performed in the MitoFish webserver (http://mitofish.aori.u-tokyo.ac.jp/, Iwasaki et al. 2013). The mitogenome map was drawn by Proksee (https://proksee.ca/), an updated version of the CGView web server (Grant and Stothard 2008).

All available 25 mitogenomes of *Rhinogobius* species in GenBank were retrieved to analyze the phylogenetic position of *R. lentiginis* in the genus (Table 1). Two *Tridentiger* species, belonging to the same Gobionellinae family, were used as an outgroup. The complete mitogenome sequences were aligned by ClustalW in MEGA X (Kumar et al. 2018). The best fit substitution model (GTR + I + G) was calculated by jModelTest 2.1.10 (Darriba et al. 2012) under the Akaike information criterion (AIC). The maximum likelihood (ML) phylogenetic tree was built with bootstrap of 1000 replications using MEGA X, and the Bayesian inference (BI) tree was reconstructed by MrBayes 3.2.6 (Ronquist et al. 2012) with two independent runs and four Markov Monte Carlo (MCMC) chains. Each run consisted of 2,000,000 generations, sampled every 100 generations with 25% of the initial trees discarded as burn-in.

**Table 1.** Species names, GenBank accession numbers, and references of all 25 mitogenomes used in phylogeny reconstruction.

Species name	Accession number	Reference
*Rhinogobius brunneus*	KT601096	–
*Rhinogobius cliffordpopei*	MK288030	Chen et al. 2019
*Rhinogobius davidi*	OM617724	–
*Rhinogobius duospilus*	MH127918	Tan et al. 2020
*Rhinogobius estrellae*	LC648295	Maeda et al. 2021
*Rhinogobius filamentosus*	OM678440	Chen et al. 2022
*Rhinogobius flumineus*	LC648306	Maeda et al. 2021
*Rhinogobius formosanus*	MN549279	–
*Rhinogobius leavelli*	MH729000	Zhang and Shen 2019
*Rhinogobius lentiginis*	OM617725	–
*Rhinogobius maculagenys*	OK545540	–
*Rhinogobius nagoyae*	LC648316	Maeda et al. 2021
*Rhinogobius niger*	OM791349	–
*Rhinogobius rubromaculatus*	KU674802	–
*Rhinogobius shennongensis*	OM961050	Shang et al. 2022
*Rhinogobius similis*	KU871066	–
*Rhinogobius* sp. MO	LC648313	Maeda et al. 2021
*Rhinogobius szechuanensis*	OM617727	Liu et al. 2023
*Rhinogobius tandikan*	LC648299	Maeda et al. 2021
*Rhinogobius wuyiensis*	OM678441	–
*Rhinogobius wuyanlingensis*	OM961051	Song et al. 2022
*Rhinogobius yaima*	LC648308	Maeda et al. 2021
*Rhinogobius yonezawai*	LC648310	Maeda et al. 2021
*Tridentiger brevispinis*	MW355489	–
*Tridentiger obscurus*	MF663787	Gong et al. 2017

## Results

### Mitogenome organization

**Figure 2.**
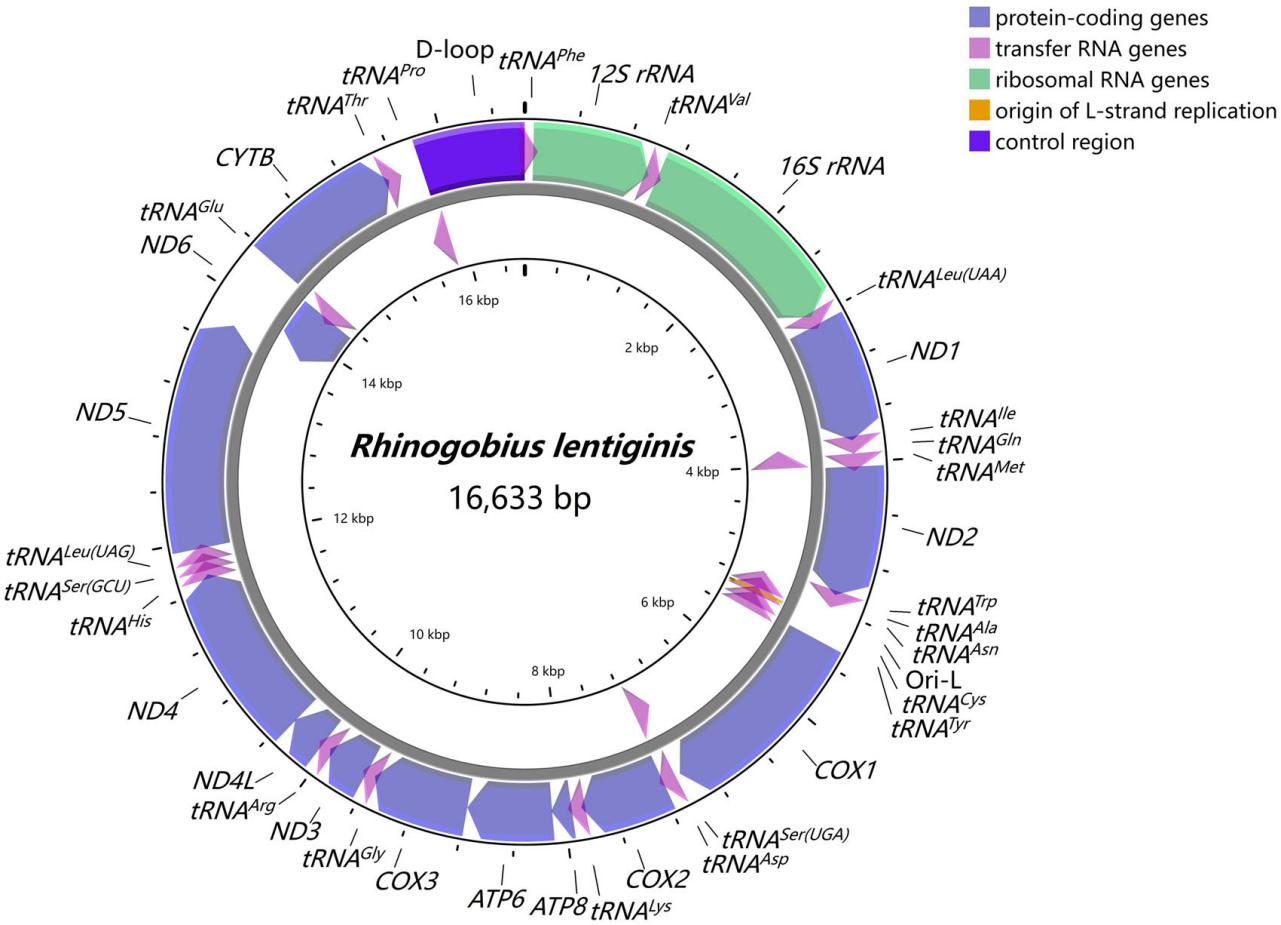
Gene map of the mitochondrial genome of *Rhinogobius lentiginis*. Genes encoded on the heavy and the light strands with inverse arrow directions were shown outside and inside the circle, respectively.

The complete mitogenome of *R. lentiginis* (GenBank accession number OM617725) was composed of 13 protein-coding genes (PCGs), 2 rRNA genes, 22 tRNA genes, and a non-coding control region (Figure 2). The length of the mitogenome was 16,633 bp, with the overall base composition of 28.44% A, 26.21% T, 16.33% G, 29.02% C. Of all 37 mitochondrial genes, 28 were encoded on the heavy strand and 9 were encoded on the light strand (*ND6*, *tRNA^Gln^*, *tRNA^Ala^*, *tRNA^Asn^*, *tRNA^Cys^*, *tRNA^Tyr^*, *tRNA^Ser(UCN)^*, *tRNA^Glu^*, and *tRNA^Pro^*). Most of the 13 PCGs contained the typical start codon ATG, except for *COX1* starting with GTG, consistent with other fish mitochondrial genomes (Tan et al. 2020; Yang et al. 2020). Conventional stop codons were observed in ten PCGs (TAA for *ND1*, *ND2*, *COX1*, *ATP8*, *ATP6*, *COX3*, *ND4L*, *ND5*, and TAG for *ND3*, *ND6*), while the incomplete stop codon T was observed in the remaining PCGs (*COX2*, *ND4*, and *CYTB*). The lengths of the PCGs varied from 165 to 1,839 bp. The *12S rRNA* (950 bp) and the *16S rRNA* (1,688 bp) were separated by *tRNA^Val^.* Total 22 tRNAs ranged from 66 bp (*tRNA^Cys^*) to 76 bp (*tRNA^Lys^*) in length. The control region of 842 bp was identified between *tRNA^Pro^* and *tRNA^Phe^*.

### Phylogenetic analysis

**Figure 3.**
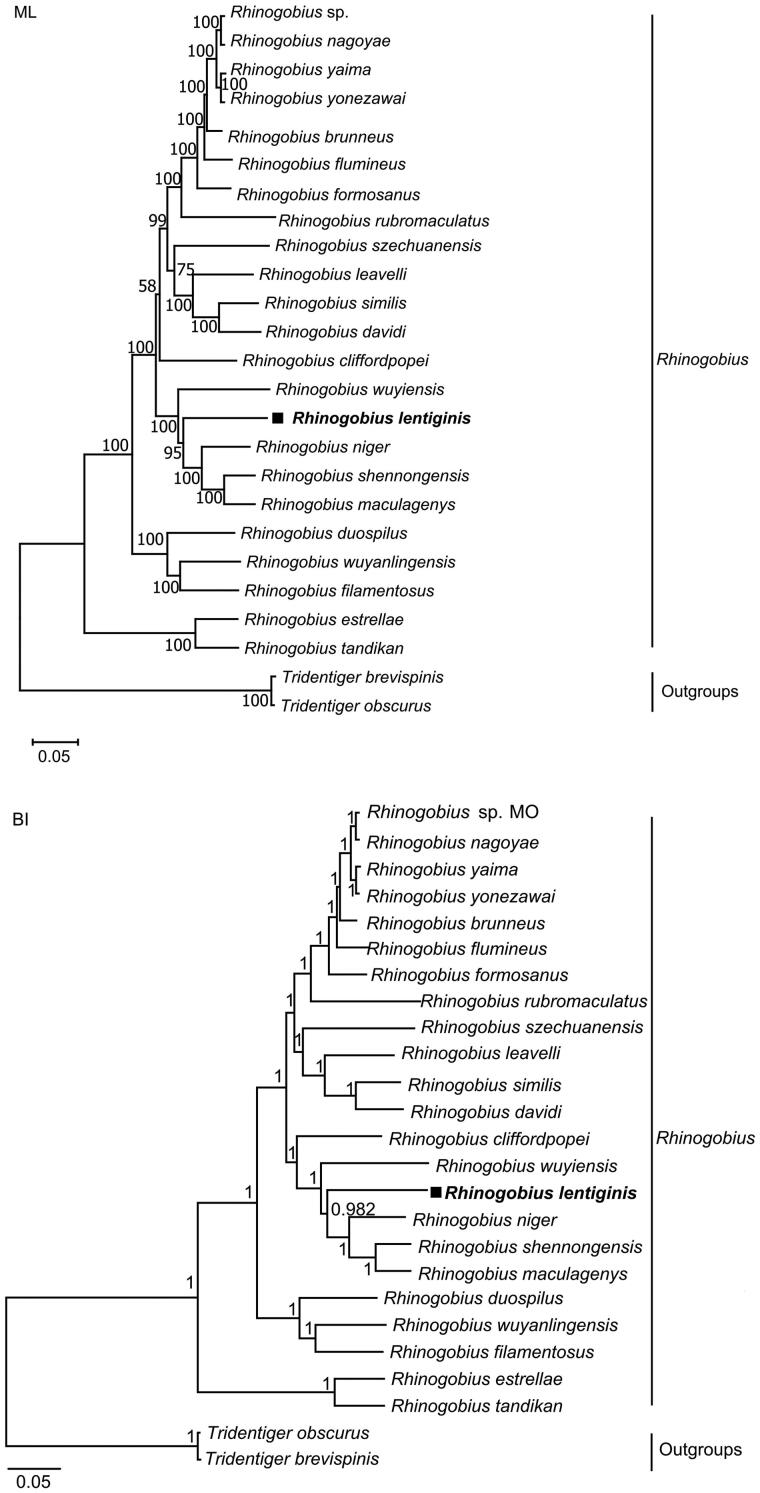
Maximum-likelihood (a) and Bayesian inference (b) phylogenetic trees inferred from complete mitochondrial genomes of *Rhinogobius lentiginis* and other 24 fishes. Numbers at nodes represent bootstrap support values for ML tree, and posterior probabilities for BI tree. The discordant result between the two trees was the placement of *R. cliffordpopei.*

Both reconstruction methods recovered that there were two main clades in *Rhinogobius* (Figure 3), in which *R. estrellae* and *R. tandikan* formed one, and other 21 *Rhinogobius* species formed the other clade. In our analyses, both trees displayed that *R. lentiginis* had a close mitochondrial genome relationship with *R. niger*, *R. shennongensis* and *R. maculagenys.* The genetic distance between *R. lentiginis* and 22 other *Rhinogobius* species ranged from 0.109 to 0.183.

## Discussion and conclusion

The complete mitogenome of the freshwater goby *R. lentiginis* had highly conserved structural organization and typical gene content, which were similar to Gobionellinae fishes (Wang et al. 2019; Yang et al. 2020). Our phylogenetic analyses of *R. lentiginis* revealed a close mitogenome relationship with *R. niger*, *R. shennongensis* and *R. maculagenys.* Present grouping results provided a better resolution for the genetic relationships among members of the *Rhinogobius* genus. Previous studies on the phylogeny of *Rhinogobius* species have shown that changes of members in the phylogenetic tree can affect the relationships of the species (Yamasaki et al. 2015; Wang et al. 2019). Therefore, further studies including morphology and genetics based on extensive taxon sampling are needed to assess the phylogenetic relationships among *Rhinogobius* species and genera. Overall, our results provide the genetic basis for resource conservation.

## Ethical approval

Experiments were performed in accordance with the recommendations of the Ethics Committee for Animal Experiments of Jiangsu Agri-Animal Husbandry Vocational College. These policies were enacted according to the Chinese Association for the Laboratory Animal Sciences and the Institutional Animal Care and Use Committee (IACUC) protocols.

## Supplementary Material

Supplemental Material

Supplemental Material

## Data Availability

The genome sequence data that support the findings of this study are openly available in GenBank of NCBI at (https://www.ncbi.nlm.nih.gov/) under the reference number OM617725. The associated ‘BioProject,’ ‘Bio-Sample’ and ‘SRA’ numbers are PRJNA808181, SAMN26031220, and SRR18131289 respectively.
